# Impact of γ-irradiation and SBR content in the compatibility of aminated (PVC/LLDPE)/ZnO for improving their AC conductivity and oil removal

**DOI:** 10.1038/s41598-022-21999-3

**Published:** 2022-11-15

**Authors:** A. I. Sharshir, S. A. Fayek, Amal F. Abd El-Gawad, M. A. Farahat, M. I. Ismail, Mohamed Mohamady Ghobashy

**Affiliations:** 1 Solid State and Accelerator Department, National Center for Radiation Research and Technology (NCRRT), Egyptian Atomic Energy Authority (EAEA), Cairo, Egypt; 2 Faculty of Engineering, Zagazig University, Zagazig, Egypt; 3 Faculty of Computers and Informatics, University Zagazig, Zagazig, Egypt; 4 Radiation Research of Polymer Chemistry Department, National Center for Radiation Research and Technology (NCRRT), Egyptian Atomic Energy Authority(EAEA), Cairo, Egypt

**Keywords:** Chemistry, Energy science and technology, Engineering, Materials science, Nanoscience and technology, Optics and photonics, Physics

## Abstract

In some cases, blends containing PVC and LLDPE show low compatibility. Adding styrene-butadiene rubber to the PVC/LLDPE mixtures leads to a noticeable increase in tensile strength and compatibility of the blends. Also, an improvement in tensile strength is observed after incorporating SBR compatibilizer resulting in entirely different gamma irradiation doses. Without a compatibilizer, the mixture exhibits a distributed PVC and LLDPE phase with variable sizes and shapes; even a sizable portion of the domains resemble droplets. Styrene butadiene rubber (SBR) and gamma radiation make mixtures of (PVC/LLDPE) more compatible. The SEM study of the blends demonstrated that adding the compatibilizer resulted in finer blend morphologies with less roughness. At the same time, gamma irradiation reduced this droplet and gave a more smooth surface. Poly(vinyl chloride) (PVC) was chemically modified with four different amino compounds, including ethylene diamine (EDA), aniline (An), p-anisidine (pA) and dimethyl aniline (DMA) for improving the electric conductivity and oil removal capability of the blend polymer. All ionomers were prepared by nucleophilic substitution in a solvent/non-solvent system under mild conditions. This work novelty shows a sustainable route for producing oil adsorption materials by recycling plastic waste. After the amination process of poly(vinyl chloride) the oil adsorption was significantly enhanced.

## Introduction

Only 18% of plastic waste is recycled and 24% is burned globally. The remaining 58% either go to landfills or are released into the environment, where plastics assemble and endure for a very long time^1^. One of the biggest worries now is how much plastic debris is making its way into the oceans. The sources of this material are closely tied to the lack of efficient infrastructure for waste management^2^. According to estimates, just 10 rivers in Asia or Africa contribute roughly 90% of the plastic waste in the ocean. A little over half of all waste polymers are made up of poly(vinyl chloride) (PVC) and polyethylene (PE)^3^. Recycling these polymers would be a desirable solution to the environmental issue, which is only worsening^4^. But choosing polymer waste of the same generic type for recycling adds a further costly step. Recycling polymer wastes together would be a desirable solution. The processing and reprocessing of PVC/PE combinations reveal many issues connected to such a process^5^. Because of the weak adhesion of the phases produced by thermodynamic incompatibility, processing PVC/PE mixtures is unlikely to give products with the required mechanical qualities^6^. There are ways to improve the situation by appropriately altering (compatibilities) chemicals that enhance the interfacial conditions between the phases^7^. As a result, the immiscible phases' interfacial energy is lowered, resulting in a finer dispersion during mixing and more excellent stability against phase separation. It is crucial to combine PVC with LLDPE, which has good heat stability and melt fluidity^8^. However, the potential solution and product properties of incompatible PVC/linear low-density polyethylene (LLDPE) mixes are undesirable due to differing structures and poor compatibility^8^.

Polymer blends have been addressed because of their theoretical and practical potential^9^. Homogenous blends, in which the components are miscible, and heterogeneous blends, in which the components are immiscible, are the two primary classifications for polymer mixtures^10^. Due to their low combinatorial entropy and high mixing enthalpy, most polymer blends are immiscible, frequently resulting in poor mechanical qualities due to the high interfacial tension and adhesion^11^. As a result, compatibilization is required for blends of immiscible polymers. By adding a third component called a "compatibilizer," the compatibilization can be forced into an incompatible binary mixture^12^. A compatibilizer is often a block or graft copolymer made of reactive polymers employed as the active interfacial component. They are predicted to function as surfactants because of their miscibility with the component polymers^13^. A standard plastic having several beneficial characteristics, including non-flammability, affordability, and formulation flexibility, is polyvinyl chloride (PVC). PVC faces challenges in expanding its application due to weak thermal stability during processing, low toughness, and a low heat-softening temperature^14^. Plasticization and improved heat-distortion use polymers are soluble in PVC. By mixing PVC, suitable polymer blends have been created^15^. One of the most popular types of synthetic rubber is styrene butadiene rubber (SBR), which has processing capabilities and physical characteristics similar to those of natural rubber (NR)^16^. SBR performs better than NR in some areas, including wear resistance, heat resistance, and aging resistance. SBR is employed here as a compatibilizer agent because of its outstanding wear resistance^17^.

In general, filling with inorganic metal oxide will likely modify all of the pure polymer's properties^18^. In fact, combining a polymer with inorganic nanoparticles creates a new material with novel physiochemical properties^18^. The characteristics of the constituent parts, such as the type of filler and polymer, determine the attributes of the final composite material^18^. These studies suggest that the shape, particle content, particle size, aggregate size, surface features, and degree of dispersion of the filler significantly impact how the filler affects the mechanical and other properties of the composites. Numerous studies have specifically examined filler content's impact on composite materials' characteristics. These nanocomposites' mechanical and electrical conductivity characteristics were closely related to the filler aspect ratio^19,20^.

Polyvinyl chloride (PVC) can be modified on its surface or molecular backbone structure depending on the nucleophilic substitution of Cl atoms. The chemical modification of PVC surface's hydrophobic behavior has various applications such as high adhesive surfaces, biomaterials scaffolds, and self-cleaning surfaces. This article suggests a novel surface modification technology of PVC reacted under gamma irradiation treatment in different doses (cross-linking reactions occur). The addition of SBR in different ratios (0, 1, 2 and 3) wt% in the blends of PVC and LLDPE via hot extruder enables the compatibility between PVC and LLDPE matrixes. The effect of SBR content on the miscibility between PVC and LLDPE will be investigated. Amination modification reactions can achieve properties adjustment of polymers (10.1016/j.jece.2017.04.024) such as PVC surface. The dehydrochlorination process, which is the basis for the chemical modification of PVC, involves the removal of Cl atoms, followed by substitution resections or the production of double bonds by the elimination of (–HCl), then addition reactions. This was accomplished by adding amine groups to the PVC in PVC/LLDPE sheets with ethylene diamine, aniline, p-anisidine and dimethyl aniline. This article demonstrated that chemical modification significantly affects the physicochemical properties of PVC/LLDPE sheets and their electric conductivity. Also discovered their oil removal ability after chemical modification compared with the blank sample of PVC/LLDPE.

## Experimental

### Materials

Recycled PVC and LLDPE powder were collected from the market without treatment. Aminating agents such as Ethylenediamine (EDA-99.5%), Aniline (An-99%), p-Ansidine (pA 99%) and N, N, Dimethylaniline (DMA 99%) were all purchased from Sigma Aldrich. PetroChina Lanzhou Petrochemical Company supplied SBR 1500E (styrene of 23.5%). From China's Shijiazhuang Zhiyi Zinc Industry Co. Ltd., zinc oxide (ZnO) was purchased.

### The manufacturing of irradiated recycled (PVC/LLDPE)-ZnO blend

The waste PVC and LDPE are soaked in silicon oil and heated for 3 h. the aim of this step is to remove any excess filler and pigment that could be presented in the waste plastic. The color of silicon oil changed, and the resultant particles were pelletized after melting. The pelletizing of PVC and LLDPE was finally dried in the oven at 50 °C for approximately 12 h. The components of PVC, LLDPE, SBR, and ZnO were manually premixed in a container; Table 1 lists the actual formulation of the samples. An extruder with twin screws was used in a melting-mixing process to create all of the blends. The temperatures from the hopper to the die were maintained at 160–290 °C, and the screw speed was kept at roughly 200 rpm. The samples were extruded and then placed in a heated mold to produce a sheet of (PVC/LLDPE)ZnO samples. The obtained sample was exposed to (0, 10 and 20) kGy of gamma radiation.

**Table 1:** represents the code of samples of amine-functionalized of PVC.

Sample no.	PVC wt%	HDPE wt %	ZnO wt%	SBR wt%	Chemical modification	Code
1	56	36	5	3	–	(PVC/LLDPE)ZnO-b
2	56	36	5	3	Ethylene diamine	(PVC/LLDPE)ZnO-EDA
3	56	36	5	3	Aniline	(PVC/LLDPE)ZnO-An
4	56	36	5	3	p-Anisidine	(PVC/LLDPE)ZnO-pA
5	56	36	5	3	Dimethyl aniline	(PVC/LLDPE)ZnO-DMA

### AC conductivity

LCR bridge model Hioki 3532 was used to measure the sample (PVC/LLDPE)/ZnO impedance Z and the phase angle between the applied AC voltage and the resulting current in the samples of irradiated and chemical modified (PVC/LLDPE)ZnO for AC conductivity measurement σAC(ω). The frequency ranged from Hz to 600 Hz. The variation in AC conductivity with a frequency at ambient temperature on an ln-ln scale. The impedance Z, sample capacitance Cp, and loss tangent Tanδ were measured using a programmable automatic 3532 LCR meter. The resistance R was parallel to all values of capacitance Cp was taken from the bridge's screen. The equation was used to calculate the total conductivity σt (w).

$$\sigma_{{\text{t}}} \left( {\text{w}} \right) = {\text{ L}}/{\text{ZA}},$$

where L is the distance between the two electrodes (the sample thickness), Z is the sample's impedance, and A is the cross-sectional area of the sample. Using the relation, the AC conductivity σAc(w) is calculated as follows:

$$\sigma_{{{\text{Ac}}}} \left( {\text{w}} \right) \, = \, \sigma_{{\text{t}}} \left( {\text{w}} \right) \, - \, \sigma_{{{\text{DC}}}} \left( {\text{w}} \right)$$

where σ_DC_(w) is termed as the DC conductivity.

### Gamma (γ) irradiation

Films were irradiated by γ- radiation with range doses (0, 10, 20) kGy, using a Co^60^ source of γ- radiation. The gamma irradiator is housed in a shielding building that is constructed upon a ground of standard density concrete (2.36 g/cc), with a thickness of about 120 cm, so that no one receives more than 10 mR of radiation during 40 h/week, or the maximum dose rate would not exceed 0.2 mR on all accessible when 1,000,000 curie cobalt radiation source is utilized.

### Simulation procedure of electric field distribution

Starting with the cable's copper core and moving outward to its outer semiconductor layer, the electric field distribution was investigated. A steady 2Uo = 24 kV 50 Hz AC power supply was applied to the cable layers (Uo is the cable's rated line to neutral voltage). The effects of electric fields were then studied using COMSOL Multiphysics. In this work, a single-core 22 kV insulated underground cable was the compression sample. The analysis uses the copper conductor with a radius of 4.165 mm, the inner semiconductor with a diameter of 4.95 mm, the insulator with a diameter of 10.45 mm, and the extrinsic semiconductor with a diameter of 11.25 mm. All radii have been calculated starting from the copper conductor's center.

### Characterization

Using an Intron mechanical testing machine, specimens' tensile characteristics in the dumbbells' shape were measured under ASTM D638 (model 5569). A 10 mm/min crosshead speed was used to measure the tensile strength and elongation. Using a Mettler Toledo 823e DSC, differential scanning calorimetry was used to measure the glass transition temperature (Tg) (Mettler Toledo International Inc., USA). In aluminum pans, samples weighing about 10 mg were preheated between 100 and 190 °C at a rate of 10 °C/min with an isothermal hold period of two minutes at 190 °C. Samples were then cooled to 100 °C once more and then heated to 100 °C at a rate of 5 °C/min. As a guide, an empty pan was used. Before the runs, liquid nitrogen was utilised to chill the samples. Infrared spectroscopy using the Fourier transform (FTIR) FTIR research was conducted utilising a Bomem-MB102 spectrometer (ABB-Bomem, USA). The spectrum was recorded between 4000 and 650 cm1 with a 4 cm-1 resolution. With a 10 kV accelerating voltage, a SEM (JSM-7500F, JEOL, Japan) was used to observe the morphology of dynamic fatigue fracture of SBR. The specimens were treated with gold spraying before being observed and then adhered to the conductive tapes. The Philips PW 1830 diffractometer was used to carry out the XRD. The X-ray beam was operated at 40 kV and 30 mA with nickel-filter Cu K (= 0.1541 nm) radiation. From 5 to 85, corresponding data were gathered in 0.02 step increments. One method is Fourier-transform infrared spectroscopy (FTIR/ATR) to produce an infrared spectrum of solid samples.

## Results and discussions

### Effect of irradiation dose and SBR content as the compatibilizer of PVC and LLDPE

This article investigated the SBR influence and gamma irradiation doses on the compatibility of PVC and LLDPE compounds. Irradiations of immiscible blend polymers are mainly compatibilized after irradiation^21^. Numerous studies have shown that γ-rays penetrate deeper and produce radicals that trigger cross-linked processes. Compared with E-Beam radiation, γ-ray has higher penetration power^22–25^. The negative charge particles of E-Beam radiation limit its penetration power^26–30^.

#### Morphological Properties of irradiated (PVC/LLDPE)ZnO blends

**Figure 1:**
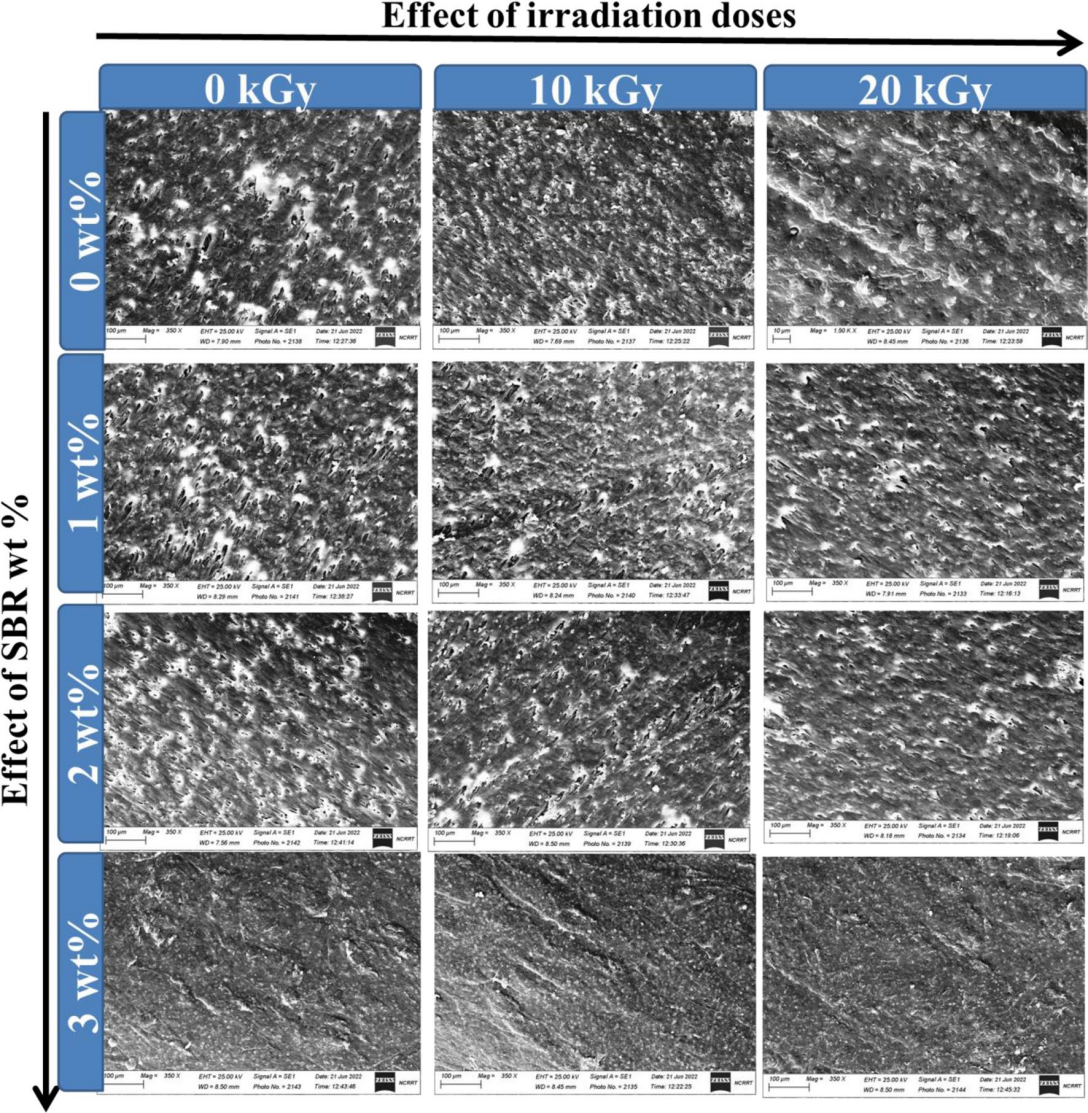
The effect of irradiation doses and SBR content in the surface morphological of (PVC/LLDPE)ZnO blends.

Figure 1 displays how the gamma radiation doses and SBR content affect the compatibility of PVC and LLDPE. Differing SBR compatibilizer concentrations produce completely distinct morphologies. Without a compatibilizer, the mixture exhibits a dispersed PVC and LDPE phase with variable sizes and shapes; even a sizable portion of the domains resemble droplets. SBR compatibilizers resulted in PVC domains with common conditions and essentially consistent measures. The compatibilizers employed have a significant impact on the PVC domain sizes. It is generally acknowledged that a compatibilizer plays two critical roles in managing a blend's morphology: coalescence prevention and interfacial tension reduction. Due to the compatibilizers' role in steric stability, it is assumed that the homogeneity of the PVC domains' size and shape generated by their addition results from a decrease in coalescence. The blank samples (0 kGy) contain more droplet matrix with more cavities, as seen in Fig. 1. The blends' SEM analysis showed that the SBR compatibilizer's addition and the irradiation method produced finer blend morphologies with less roughness. In PVC/LLDPE blends, the SBR compatibilizer reduced droplet coalescence and aided in stabilising fine morphology. The compatibility between LLDPE and PVC matrixes was significantly improved by the SBR compatibilizer level of 3 wt%. Additionally, gamma irradiation slightly improves compatibility rather than just the SBR impact. The coalescence of recently produced droplets becomes more significant when SBR concentration increases.

#### Mechanical properties of irradiated (PVC/LLDPE)ZnO blends

**Figure 2:**
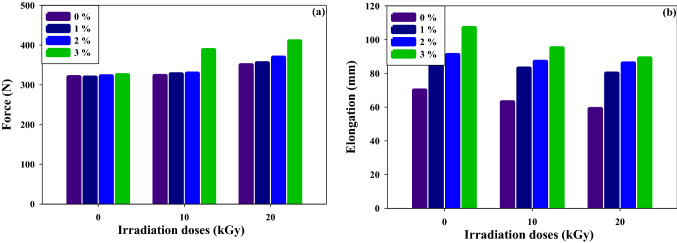
The effect of SBR content and irradiation doses on the mechanical properties of (PVC/LLDPE)ZnO blend.

Due to their low interphase adhesion and high interfacial tension, PVC and LLDPE form incompatible combinations, as reported in several academic publications^31–33^. The mechanical properties of PVC and LLDPE are almost inferior^33^ and can be improved by the SBR addition and gamma irradiation process. Figure 2 demonstrates how the SBR and irradiation processes improve the blends' tensile strength and elongation. This result is due to the increased compatibility of PVC with LLDPE. Based on ASTM standards, a stress test was plotted in Fig. 2a on different contents of SBR compatibilizer and gamma irradiation doses. The force strength of (PVC/LLDPE)ZnO samples is enhanced after adding SBR and exposure to gamma radiation than blank samples. The compatibilizer (SBR) has a good effect on interfacial bonding after the irradiation process and increases the force strength of the (PVC/LLDPE)ZnO blends. The addition of SBR increases the force strength and exhibits superior material strength after gamma irradiation due to gamma irradiation-induced crosslinking of SBR^34^. In Fig. 2b the elongation (mm) is increase about 21%, 30% and 52% with SBR content increase from 1%, 2% and 3% at 0 kGy, respectively. This difference is due to the SBR chains made plasticizer effect in blends sample. Based on the definition of plasticization, the elongation should increase with an increase in the plasticizer concentration^35–38^. After the gamma irradiation process, the elongation is decreased. For example, at 3% SBR the elongation decreased from 11 to 16.8% for irradiation doses at 10 kGy and 20 kGy, respectively. This due to the gamma irradiation induced further cross-linked reactions and so decreased the movement of chains and elongation^39,40^.

### Amine-functionalized of PVC in irradiated (PVC/LLDPE)/ZnO blend

**Figure 3:**
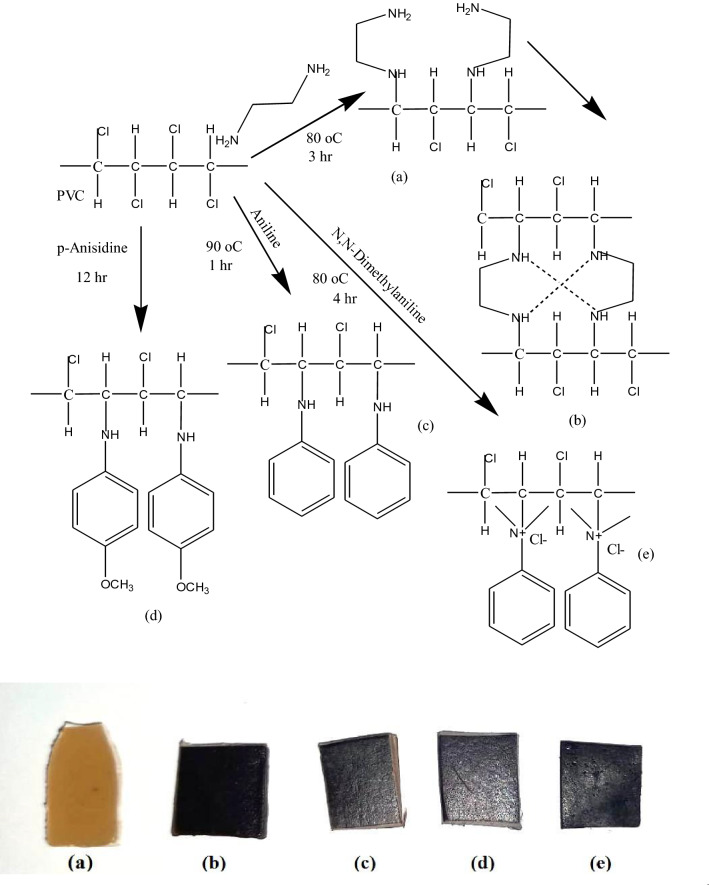
proposed amine-functionalized poly (vinylchloride/Linear low-density polyethylene)/ZnO with the selected different four amines; (**a**) ethylene diamine EDA, (**c**) aniline An, (**d**) p-anisidine pA, (**e**) N, N dimethyl aniline DMA and (**b**) a possible crosslinking mechanism for two products functionalized by diamine precursors. The photo in bottom is show the changing of samples color after amination reaction (**a**) (PVC/LLDPE)ZnO-b, (**b**) (PVC/LLDPE)ZnO-EDA, (**c**) (PVC/LLDPE)ZnO-An (**d**) (PVC/LLDPE)ZnO-pA and (**e**) (PVC/LLDPE)ZnO-DMA.

Poly(vinyl chloride) (PVC) was chemically modified with four different amino compounds, including ethylene diamine (EDA), aniline (An), p-anisidine (pA) and dimethyl aniline (DMA) for improving the electric conductivity and oil removal capability of the blend polymer. The chemical structure and the proposed mechanism of PVC amination are represented in Fig. 3. After amination reactions, all samples of modified (PVC/LLDPE)ZnO-b exhibit coloration degree varying from brown to dark brown. All ionomers were prepared by nucleophilic substitution in a solvent/non-solvent system at mild conditions (Table 1). Chemical modification of PVC by nucleophiles was conducted in a 100-ml three-neck round-bottomed flask equipped with a magnetic stirrer and condenser. Practically, 90 v% of ethylene diamine (EDA) dissolved in ethanol was added dropwise to 5 g (PVC/LLDPE)/ZnO strip soaked in 20 ml of ethanol solutions. The mixture was heated to 80 °C for 3 h and then the modified (PVC/LLDPE)/ZnO strip was immersed in a mix of ice and water at the thermal equilibrium. The new modified (PVC/LLDPE)/ZnO-EDA was obtained and kept in dry conditions for further experimental studies. A mixture of 5 g of (PVC/LLDPE)/ZnO and 10 ml of aniline dissolved in10 ml of ethanol solvent was stirred for about an hour and at a temperature of 90 °C. After that, the aniline modified (PVC/LLDPE)/ZnO was washed with a mixture of ethanol/water. For the modified (PVC/LLDPE)/ZnO with p-Anisidine, a reaction mixture of 5 g and 10 g, respectively, was continued by stirring in 10 ml of ethanol for about 12 h at room temperature. The resulting modified strip polymer (PVC/LLDPE)/ZnO-pA was washed and dried. A mixture of dimethyl aniline (30 ml) dissolved in 10 ml of ethanol containing 5 gm of (PVC/LLDPE)/ZnO strip. The mixture was refluxed in 100-ml three-neck round-bottomed flask for 4 h at a temperature of 80 °C. The obtained (PVC/LLDPE)/ZnO-DMA was washed several times with an ethanol/water mixture. Figure 3 shows the proposed mechanism reactions of Poly(vinyl chloride) (PVC) with four different amino compounds, including ethylene diamine (EDA), aniline (An), p-anisidine (pA) and dimethyl aniline (DMA). The functionalized amine PVC was formed by the nucleophilic attack of the (N) atom on the carbon-bearing chlorine atom in the polymeric chain of PVC, starting the process. The chloride anion was left as a good leaving group (-HCl).

#### Chemical modification performed by FTIR/ATR

**Figure 4:**
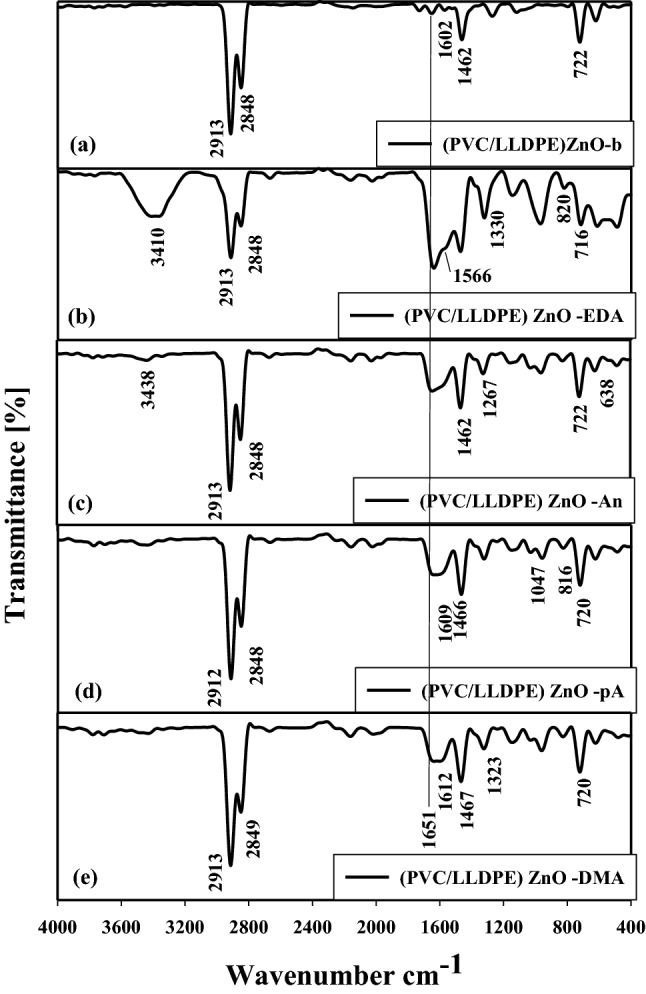
The FTIR/ATR curves for amined modified PVC in (PVC/LLDPE).

After the facile chemical modification by amination groups of PVC molecules in (PVC/LLDPE)ZnO-b via the nucleophilic chemical reactions. Figure 4 shows the FTIR curves of a new amine functionalized PVC group formed on the (PVC/LLDPE)ZnO-b samples. The chemical structure of the modified (PVC/LLDPE)ZnO was performed by FTIR, and each of the modified (PVC/LLDPE)ZnO showed its characteristic FTIR peaks according to its chemical structure. The successful modification of PVC with four different amines can be demonstrated by FTIR spectroscopy in Fig. 4. As shown in Fig. 4a (PVC/LLDPE)ZnO-b cross-linked reactions induced by gamma irradiations take place mainly due to the radical formations and elimination of chloride ions (e.g., –HCl)^41^. This dechlorination process is responsible for forming short polymeric chains containing C=O and –CH=CH– moieties. As irradiation of (PVC/LLDPE)ZnO-b progresses, the formation of the C=O and –CH=CH– becomes noticeably significant. Therefore, FTIR spectroscopy was used to examine the growth of the absorption peaks corresponding to the C=O and –CH=CH– groups located at 1651 cm^-1^ and 1602 cm^-1^, respectively. The changes in the position of C=O and C=C for the (PVC/LLDPE)ZnO after chemical modification are predicted as shown in Figs. 4b-e. It was clear that the changes in the C=O and C=C were significantly more significant and sharper in the case of the (PVC/LLDPE)ZnO-b. In addition, the two FTIR peaks at 2913 cm^-1^ and 2884 cm^-1^ correspond to the asymmetric and symmetric stretching vibration of –CH– is a repeat unit in LLDPE and PVC molecules. The FTIR peaks at 722 cm^-1^ and 1462 cm^-1^ correspond to the stretching vibration of C–Cl and the bending vibration of –CH_2_ in PVC and LLDPE molecules, respectively. Figure 4b shows the FTIR curve of (PVC/LLDPE)ZnO–EDA sample, the characteristic two peaks located at 1566 cm^-1^ and 820 cm^-1^ are assigned to bending vibration of (N–H) and (C–N) bonds, respectively. The peak located at 1330 cm^-1^ corresponds to the stretching vibration of (C–N) bonds in PVC/LLDPE)ZnO-EDA sample. In addition, the broad beak located at 3410 cm^-1^ corresponds to the starching vibration of (N–H) in primary amine. The broadening peak of 3410 cm^-1^ is due to the intramolecular hydrogen bonds excited between NH_2_ groups, as represented in Fig. 3b. Also, the broad peak of 3410 cm^-1^ confirms the in situ cross-linked reactions take placed in PVC/LLDPE)ZnO-EDA sample. Figure 4c shows the characteristic peaks of aniline molecules in (PVC/LLDPE) ZnO-An sample. The two FTIR peaks located at 3438 cm^-1^ and 1267 cm^-1^ correspond to the stretching vibration of (N–H) and (C–N) bonds in secondary amines due to the reaction between aniline and PVC molecules in (PVC/LLDPE) ZnO-An sample. Figure 4d shows the characteristic FTIR peaks of (PVC/LLDPE)ZnO-pA located at 1609 cm^-1^ and 1047 cm^-1^ that assignment to the stretching and bending vibration of C–O and N–H bonds in p-anisidine molecules. Figure 4e show the characteristic FTIR peaks of (PVC/LLDPE) ZnO -DMA located at 1223 cm^-1^ that assignment to the stretching vibration of C-N of tertiary amine and 1612 cm^-1^ that that assignment to the stretching vibration of C=C of a benzene ring with no NH peak observed for dimethyl aniline molecules.

#### The XRD analysis of modified (PVC/LLDPE)ZnO

**Figure 5:**
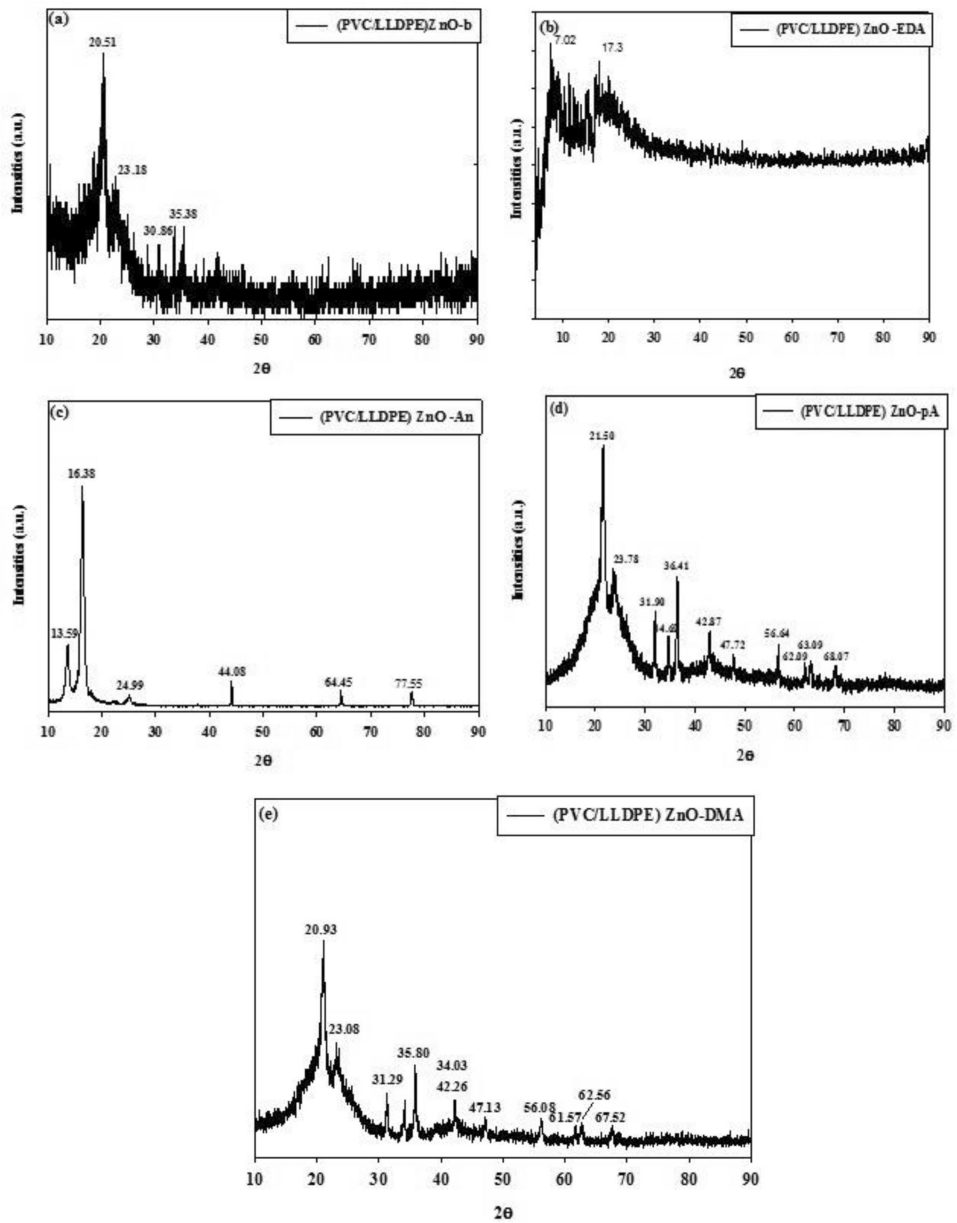
The XRD curves of aminated mopdfied PVC in (PVC/LLDPE)ZnO samples.

Figure 5a shows the XRD pattern of virgin LLDPE in the blank sample of (PVC/LLDPE)ZnO-b has appeared at 20.51° and 23.18°, which are assigned to the 110 and 200 reflections of LLDPE. At the same time, the XRD pattern of virgin PVC film at 2θ ~ 17° and 26° does not appear. This could be due to the gamma irradiation that may cause dechlorination (–HCl) of PVC molecules^42^. The addition of ZnO nanoparticles into (PVC/LLDPE) matrix provided new XRD peaks located at 30.86°, 34.4°, 36.3°, 47.51°, 56.61°, 62.81°, 66.41°, 67.91°, 69.10°, 72.52°, 76.9° that indexed to ZnO pattern of (100), (002), (101), (102), (110), (103), (200), (112), (201), (004) and (202). Figure 5b–e shows the XRD pattern of (PVC/LLDPE)ZnO-b modified chemically by the nucleophilic substitution process. Compared to Fig. 1a, the intensities of the diffraction peaks significantly changed after chemical modification. Figure 3b shows the XRD peaks of (PVC/LLDPE) ZnO –EDA sample that almost exhibit only two peaks at 7.02° and 17.3°. Figure 5c shows the XRD peaks of (PVC/LLDPE)ZnO-An, which show the sharp characteristic peaks of aniline molecules with high crystallinity at 16.38°^29^. Figure 5c,d shows the XRD peaks of both (PVC/LLDPE)ZnO–pA, (PVC/LLDPE)ZnO-DMA that exhibit the shifted peaks of LLDPE and ZnO pattern.

#### Thermal properties of modified (PVC/LLDPE)ZnO performed by DSC

**Figure 6:**
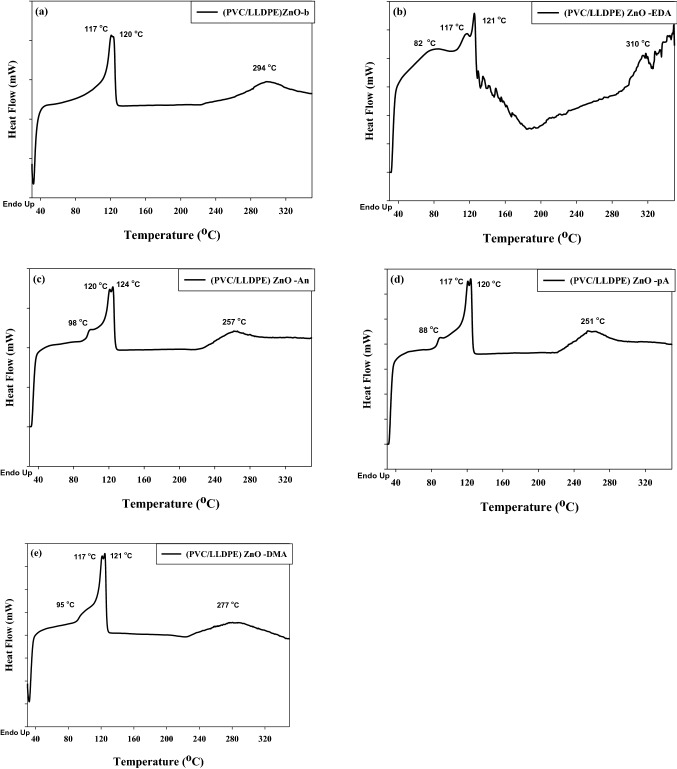
The DSC thermograms of aminated modified PVC in (PVC/LLDPE)ZnO.

Differential scanning calorimetry was performed to determine the melting transition temperature (*T*_*m*_) and glass transition temperature (*T*_*g*_) of (PVC/LLDPE)ZnO-b recycled blend and their chemical modification matrices. The two temperature is an essential parameter in polymer characterization to evaluate the compatibilizing agent effect of SBR added to (PVC/LLDPE)ZnO-b sample besides the chemical modification. Figure 6 shows the measurement DSC of modified (PVC/LLDPE)ZnO samples, Compare to the blank sample, the four DSC curves of modified samples exhibited changes in the melting point of PVC. The obtained results is confirmed that the amination reactions take place in PVC molecules by dechlorination. Figure 6a exhibit three melting points (*T*_*m*_) of irradiated (PVC/LLDPE)ZnO-b sample, two melting points for LLDPE at the temperature of 117 °C, 120 °C and 294 °C assignment to PVC molecules. The melting peak of LLDPE into double peaks located at 117 °C, 120 °C of the irradiated samples is due to the presence of both irradiated and non-irradiated regions that give different melting temperatures. The highest melting point (*T*_*m*_) at 120 °C is due to gamma irradiation-induced cross-linked reactions of LLDPE. As expected, the glass transition temperature (*T*_*g*_) of (PVC) at ~ 85–93 °C disappeared. This could be due to the effect of compatibilizing agent (SBR) as a plasticizer, limiting the glassy temperature of PVC^43^. Figure 6b shows the DSC curve of (PVC/LLDPE) ZnO –EDA sample that exhibited a peak abroad at 82 °C, corresponding to the moisture content. The results indicated that amination reactions by EDA is an effective method to increase the hydrophilicity of PVC. In addition, the increased melting point (310 °C) of (PVC/LLDPE) ZnO–EDA sample is due to the intramolecular H bonds established after chemical modification as confirmed by FTIR data. Figure 4 c-d exhibit the DSC of the three ((PVC/LLDPE)ZnO-An, (PVC/LLDPE) ZnO-pA and (PVC/LLDPE)ZnO-DMA)) samples with single Tg at the temperature of (98, 88 and 95) °C, respectively. PVC has Tg at a temperature of 93.5 °C according to the DSC data found in the literature^44–46^. This fact reinforces the plasticizing effect induced by p-anisidine, especially the percent of 5 w% of ZnO it could increase the Tg when it acts as a filler^47,48^. Furthermore, when aniline and N, N, dimethyl aniline were reacted with PVC molecules caused an increase in their *Tg* due to the restriction on the free rotation and hence restricted segmental motion^49^. On the other hand, the melting point (*T*_*m*_) of PVC was decreased at the temperature of 257 °C, 251 °C and 277°C for ((PVC/LLDPE)ZnO-An, (PVC/LLDPE) ZnO-pA and (PVC/LLDPE)ZnO-DMA)) samples, respectively. The DSC data confirmed the chemical amination modification of (PVC/LLDPE)ZnO-b samples.

### The role of the aminated modified PVC/LLDPE on the oil removal capacity

**Figure 7:**
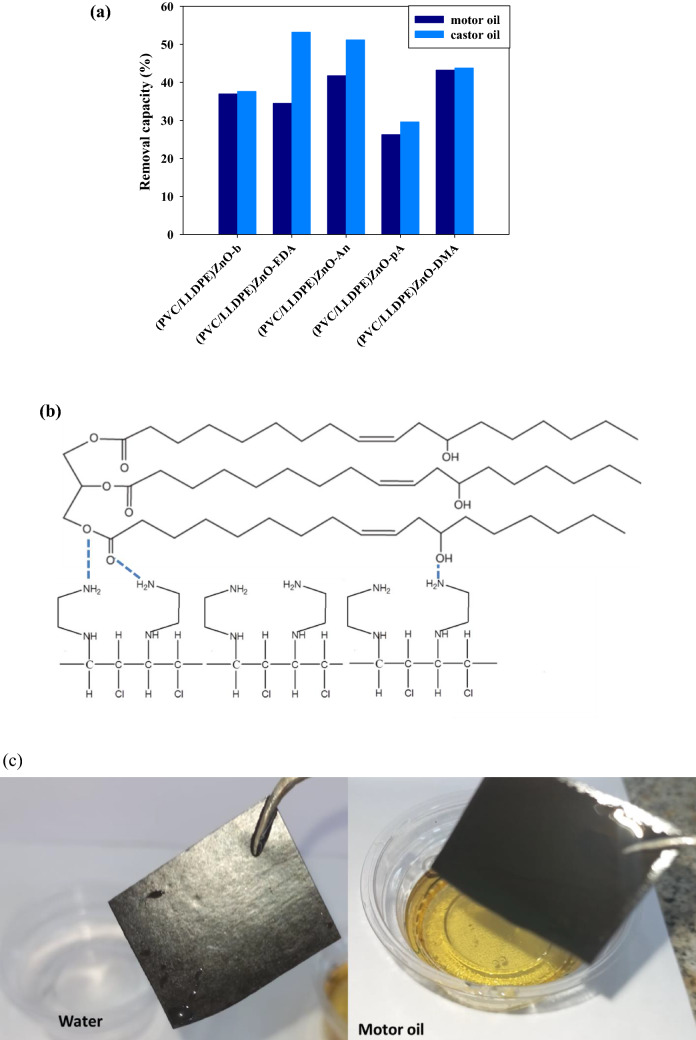
(**a**) adsorption capacity of motor oil and castor oil by aminated modified PVC in (PVC/LLDPE)ZnO depending on the nature of the aminating reagent, (**b**) the proposed intermolecular H-bonds formation between Castro oil and NH_2_ groups in (PVC/LLDPE)ZnO-EDA samples and (**c**) the efficiency of motor oil removal by (PVC/LLDPE)ZnO-DMA.

Among the aminating groups that were used, aniline (An), p-anisidine and dimethyl aniline (DMA) appear to possess the best motor oil removal capacity (41, 38 and 43) %, respectively. The obtained data confirmed that the three aminating reagents (An, pA and DMA) become more motor oil removal effective than the blank sample due to the percent of the aromatic benzene ring. The two samples of (PVC/LLDPE)ZnO-b and (PVC/LLDPE)ZnO-EDA seem to be the least effective for this task due to the absence of aromatic benzene ring and extra NH_2_ group in (PVC/LLDPE)ZnO-EDA sample. A decrease in removal capacity of motor oil than castor oil was observed for all samples due to the adsorption of castor oil on amine groups due to the formation of intermolecular H bonds. As shown in Fig. 7a the castor oil removal capacity of the blank sample (PVC/LLDPE)ZnO-b modified by three amine reagents (EDA, An and DMA) was enhanced. (PVC/LLDPE)ZnO-EDA sample and two other aminated modified samples of (PVC/LLDPE)ZnO-An and (PVC/LLDPE)ZnO-DMA showed high removal capacity of castor oil (53, 51 and 43) %, respectively. The structural data of castor oil and aminated modified (PVC/LLDPE)ZnO-EDA sample prove the high adsorption capacity due to the intermolecular H-bonds established between C=O and OH groups in castor oils with NH_2_ groups in (PVC/LLDPE)ZnO-EDA as summarized in Fig. 7b. All of these modified structural parameters seem to be influenced by oil adsorption capacity. This highlights that the distribution of amine sites on the adsorbents is critical to high castor oil adsorption performance. Figure 7c highlights that the aromatic benzene ring in (PVC/LLDPE)ZnO-DMA gives hydrophobic sites on the surface of the adsorbent is critical to the high performance of motor oil adsorption and water repellent.

### The electric conductivity of aminated modified PVC

**Figure 8:**
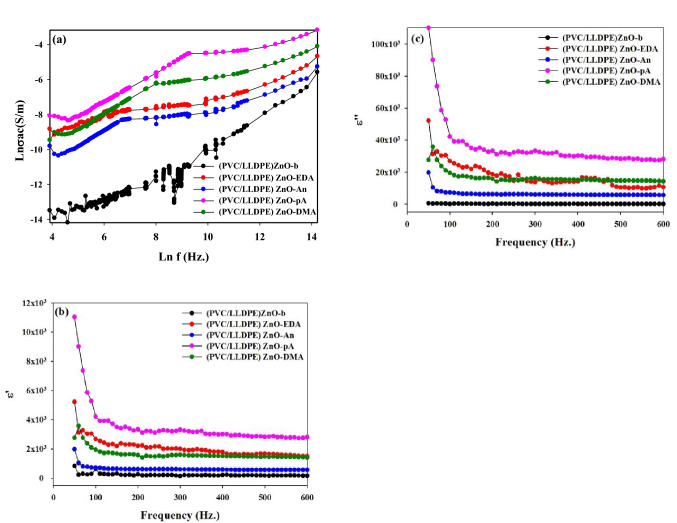
(**a**) The AC electric conductivity, (**b**) permittivity εʹ and (**c**) the dielectric loss εʺ at room temperature ~ 25 °C of aminated modified PVC as a function of frequency.

Figure 8a represents the electrical conductivity irradiated blank (PVC/LLDPE)ZnO-b and modified aminated sample. It is observed that the (PVC/LLDPE)ZnO-pA blend had higher electrical conductivity than other samples at any given frequency despite the increased electrical conductivity of the PVC with the increase in frequency. The increase in the modfied blend's conductivity could be attributed to the formation of amine sites which may be in the protonation state. The protonation is chemically formed intermolecular and intramolecular H-bonds as an inorganic doping process: the protonated of amine groups is known to be more conducting due to a high degree of conjugation. The permittivity ε' and dielectric loss ε" for aminated modified PVC samples were represented in Fig. 8b,c over a frequency range 0.01 Hz up to 600 Hz. The measurements were carried out at room temperature (25 ± 1 °C). From Fig. 8b,c, the values of ε' decrease by increasing the applied frequency showing an anomalous dispersion. In such a range, the permittivity has a contribution from orientation polarization. Also, ε' & ε" increase in order (PVC/LLDPE)ZnO-pA, (PVC/LLDPE)ZnO-EDA, (PVC/LLDPE)ZnO-DMA, (PVC/LLDPE)ZnO-An and (PVC/LLDPE)ZnO-b. This increase in ε' & ε" with the incorporation amino groups is due to the rise in dipoles-dipoles interactions and intramolecular H bonds which leads to an increase in the orientation polarization and also to the presence of interfacial polarization. The blend plastic used to manufacture medium voltage cables has many advantages like a low dissipation factor of about 0.03% at 20 °C, a low dielectric constant of 2.2–2.5, good thermo-mechanical properties and a high operating temperature of about 90 °C^50^. The aged samples show increasing dielectric constant and dielectric loss^51^. In our samples, dielectric loss is more minor for neat samples (PVC/LLDPE)ZnO-b. After chemical modification, the dielectric loss is increased without the aging process. It means that the existence of nanoparticles^52^ and amine sites on the sample blend will increase dielectric loss. Therefore it is possible to postulate that chemical modification will increase the dielectric constant and dielectric loss.

### Simulation and modeling of electric field distribution of aminated modified PVC

**Figure 9:**
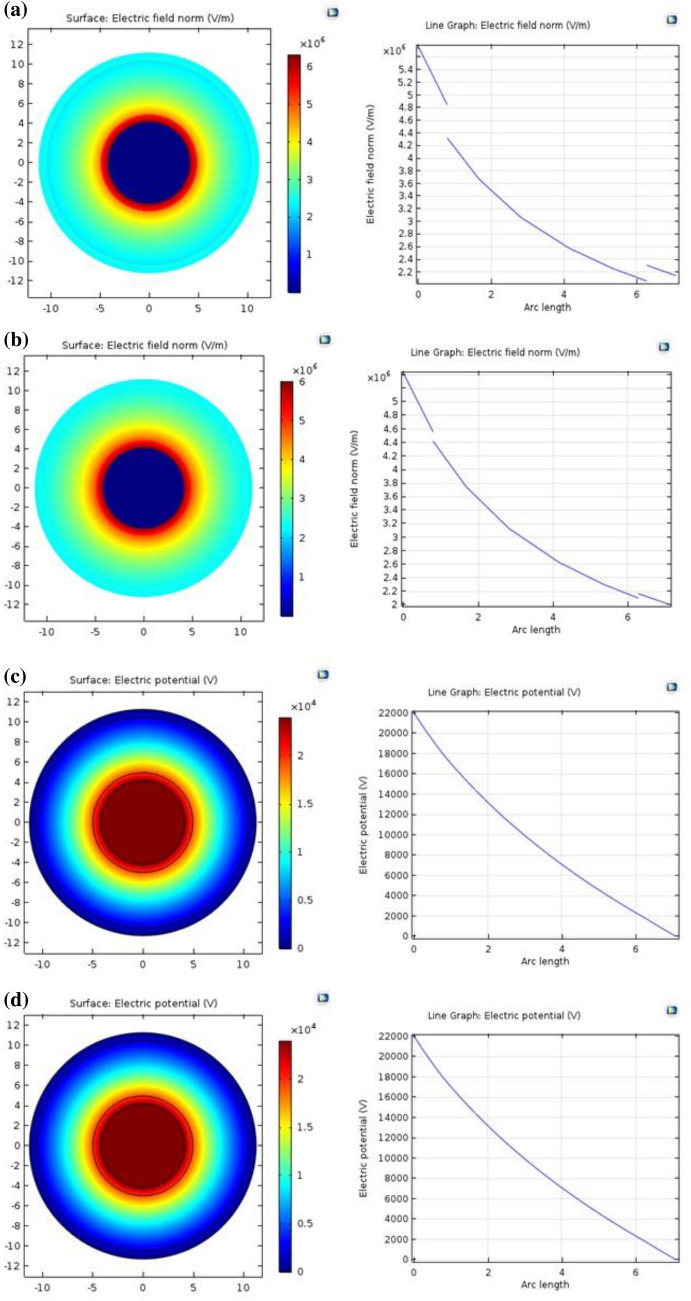
Electric field distribution in medium-voltage cable of (**a**) (PVC/LLDPE)ZnO-b. (**b**) (PVC/LLDPE)ZnO-pA. (**c**) (PVC/LLDPE)ZnO-b. (**d**) (PVC/LLDPE)ZnO-pA sample.

The cable was a single core 22 kV shielded underground medium voltage cable. COMSOL Multiphysics was used to simulate the electric field distribution in Medium Voltage Cables in this study. The electric field distribution has been studied, starting from the copper core to the outer semiconductor layer of the cable. Figure 9a shows that at 1 mm of arc length, the distribution of the electric field inside (PVC/LLDPE)ZnO-b sample marked cables is non-uniform. For (PVC/LLDPE)ZnO-pA sample, as shown in Fig. 9b the electric field distribution is starting to become uniform and gradually decreases from the inside to the outside. This is because the p-anisidine filled (PVC/LLDPE)ZnO-pA sample preserves a uniform electrical field and reduces electrostatic tension, increasing relative permittivity values for inner semiconductors and outer semiconductors from 2.05 to 2.23. The optimum AC conductivity (AC: 2.44 × 10^–4^ S/m) in minimum relative permittivity (2.23) was achieved for (PVC/LLDPE)ZnO-pA irradiated at 20 kGy. Figure 9c,d demonstrate the electric potential distribution of (PVC/LLDPE)ZnO-b and (PVC/LLDPE)ZnO-pA samples, respectively with no change noted in the behavior curve. The electric potential distribution gradually decreases for two samples from 22,000 to 0 V.

## Conclusions

The amination of a non-functional PVC matrix was performed using a simple chemical reaction process between four different amine reagents and PVC to develop an adsorbent for oil removal from aqueous phases. PVC and LLDPE blends were successfully compatibilized by SBR with ionizing irradiation, namely gamma irradiation in the dose of 0, 10 and 20 kGy. The tensile and stress tests indicated a remarkable improvement in mechanical properties after the gamma irradiation process with increasing SBR content. In particular, the elongation (mm) is increase about 21%, 30% and 52% with SBR content increase from 1%, 2% and 3% at 0 kGy, respectively. The use of SBR prevents the aggregate formation and the ZnO domain is a remarkably uniform distribution, thus indicating a better particle dispersion. The FT-IR and XRD results confirm the aminated characteristics of the (PVC/LLDPE)ZnO. DSC revealed decreased Tg of PVC and a decrease in their melting points with the degree of crystallinity of (PVC/LLDPE)ZnO blends due to the formation of less perfect crystals resulting from the amination modification of PVC. At the same time, the melting point of LLDPE is split into two peaks due to the presence of both irradiated and non-irradiated regions, providing two LLDPE regions. The modified blends show a significant enhancement of oil removal compared to the unmodified sample. The electrical conductivity of modified blends increases with the frequency. The increase in electrical conductivity of the target polymer, PVC, is mostly due to the dehydrochlorination process (Supplementary Video S1).

## Supplementary Information

Supplementary Video 1.

## Data Availability

The data that support the findings of this study are available from the corresponding author upon reasonable request.
