# Author Correction: Impact of γ-irradiation and SBR content in the compatibility of aminated (PVC/LLDPE)/ZnO for improving their AC conductivity and oil removal

**DOI:** 10.1038/s41598-022-26900-w

**Published:** 2022-12-29

**Authors:** A. I. Sharshir, S. A. Fayek, Amal F. Abd El-Gawad, M. A. Farahat, M. I. Ismail, Mohamed Mohamady Ghobashy

**Affiliations:** 1 Solid State and Accelerator Department, National Center for Radiation Research and Technology (NCRRT), Egyptian Atomic Energy Authority (EAEA), Cairo, Egypt; 2 Faculty of Engineering, Zagazig University, Zagazig, Egypt; 3 Faculty of Computers and Informatics, University Zagazig, Zagazig, Egypt; 4 Radiation Research of Polymer Chemistry Department, National Center for Radiation Research and Technology (NCRRT), Egyptian Atomic Energy Authority(EAEA), Cairo, Egypt

Correction to: *Scientific Reports* 10.1038/s41598-022-21999-3, published online 15 November 2022

The original version of this Article contained an error in the name of the author A. I. Sharshir which was incorrectly given as Ahmed Ibrahim Sharshir.

The original Article has been corrected.

